# A case of cardiac arrest due to postpartum hemorrhage treated with hysterectomy and extracorporeal membrane oxygenation

**DOI:** 10.1002/ccr3.7554

**Published:** 2023-06-13

**Authors:** Naoki Tsuchiya, Soichiro Obata, Michi Kasai, Etsuko Miyagi, Shigeru Aoki

**Affiliations:** ^1^ Perinatal Center for Maternity and Neonates Yokohama City University Medical Center Yokohama Japan; ^2^ Department of Obstetrics and Gynecology Yokohama City University Hospital Yokohama Japan

**Keywords:** disseminated intravascular coagulation, extracorporeal membrane oxygenation, heart arrest, postpartum hemorrhage, respiratory insufficiency

## Abstract

Although extracorporeal membrane oxygenation is relatively contraindicated in patients with severe disseminated intravascular coagulation (DIC), it can be safely introduced by providing adequate anti‐DIC therapy.

## Graphical abstract

A uterine rupture caused massive postpartum hemorrhage and disseminated intravascular coagulation which resulted in maternal cardiac arrest.

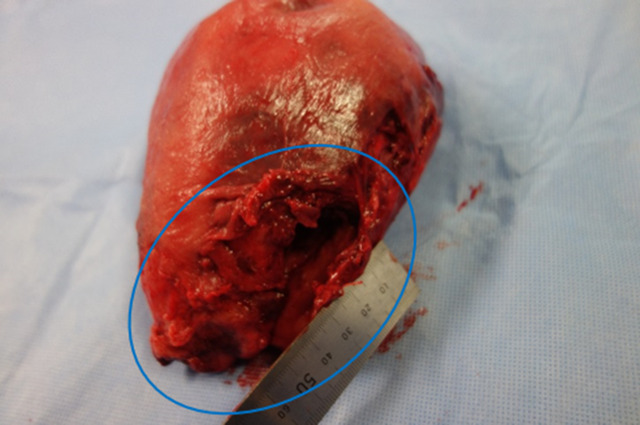

## 1 INTRODUCTION

There are various causes of maternal cardiac arrest, including hemorrhage, heart failure, amniotic fluid embolism, and sepsis. It is necessary to identify the cause of maternal cardiac arrest based on the symptoms and course of delivery and to manage the cause immediately.^1^ Postpartum hemorrhage (PPH) is the most frequent cause of maternal cardiac arrest. PPH, which is severe enough to cause cardiac arrest, is likely to be complicated by disseminated intravascular coagulation (DIC), which requires intensive management.

Extracorporeal membrane oxygenation (ECMO) is a life‐support for respiratory function that is introduced for severe respiratory and circulatory failure and following resuscitation in cardiopulmonary arrest.^2^ Currently, the adoption of ECMO is expanding because of technological innovations, and it is expected that the number of cases that use ECMO will soon increase.^2^, ^3^ In the perinatal field, good perinatal outcomes have been reported in patients with acute respiratory distress syndrome (ARDS), pulmonary embolism, perinatal cardiomyopathy, and infectious diseases after the introduction of ECMO.^4^ However, the introduction of ECMO is relatively contraindicated in patients with DIC^5^, and there are no reports on the introduction of ECMO in PPH patients with DIC.

In this report, we describe a case of respiratory failure after resuscitation from cardiac arrest due to PPH complicated with DIC. This patient was successfully treated by the introduction of ECMO without any complications due to the use sufficient anti‐DIC therapy.

## 2 CASE PRESENTATION

The patient was 41‐year‐old, gravida 4, para 1 (one vaginal delivery and 2 artificial abortions) woman. She had a history of panic disorder, but took no medication, and had no other complications. After spontaneous conception, she underwent a perinatal checkup at an obstetric clinic from the early gestational weeks, and there were no problems during her pregnancy course.

Spontaneous labor was initiated at 40 weeks and 4 days of gestation, and she was admitted to the hospital. At 40 weeks and 5 days of gestation, cervical dilation did not progress beyond 7 cm; thus, labor induction with oxytocin was initiated to promote labor. Cardiotocography (CTG) showed tachysystole of the uterus, and the cervical dilation was fully open 65 minutes after the start of induction. Because the CTG trace showed recurrent severe variable deceleration (bottom 60 bpm, 30–45 s), the infant was delivered by forceps assisted with uterine fundal pressure. She delivered a baby weighing 3620 g, with an Apgar score of 9/9 (1/5 min).

After delivery, she became disquieted and her shock index (SI) was greater than 1.2. Vaginal bleeding was of normal volume, but intra‐abdominal bleeding was suspected because ultrasonography findings detected fluid storage in the peritoneal cavity. We received a request to transport her to our hospital 32 min after delivery, and transport from the previous hospital was started 60 min after delivery; she arrived at our hospital 83 min after delivery.

**FIGURE 1:**
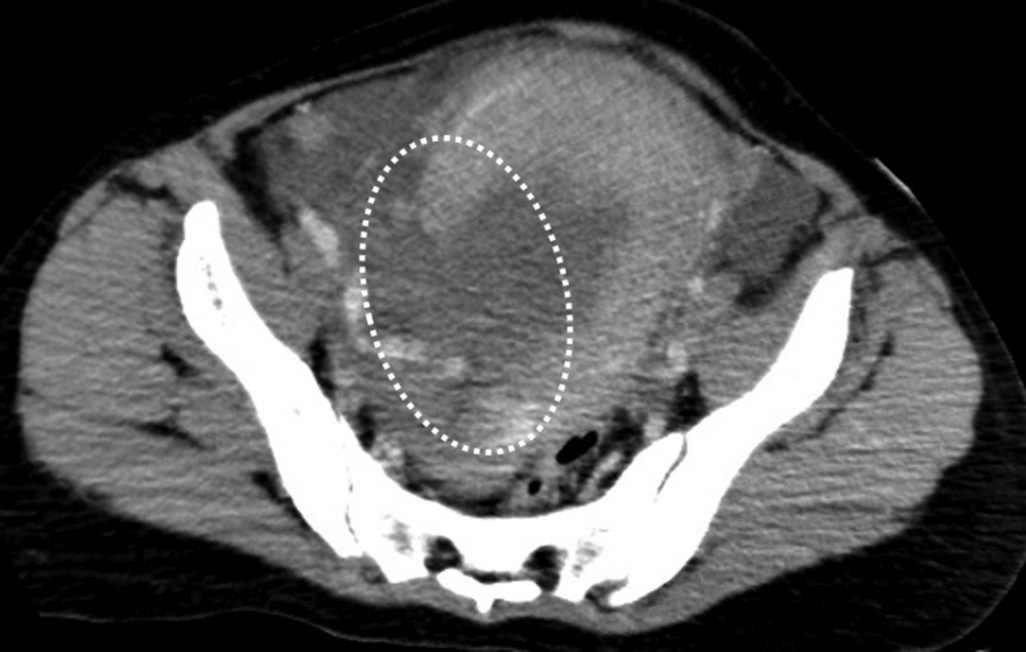
Contrast‐enhanced computed tomography axial image at the time of arrival. This image shows separation of the uterine wall and leakage of the contrast medium from the cervix to the right side of the uterine corpus.

We assessed the patient in the Emergency Department, where computed tomography (CT) could be performed. On arrival, her Glasgow Coma Scale score was 13 (E3V4M6), and she was delirious. Due to her high SI (1.1, BP: 142/92 mmHg, HR: 163 beats/min, regular rhythm) and continuous non‐coagulable vaginal bleeding, rapid blood transfusion (red blood cells [RBC], fresh‐frozen plasma [FFP], and fibrinogen)was started. Abdominal ultrasound showed a large amount of fluid retention in the intraperitoneal space and contrast‐enhanced CT (Figure 1), showing tears in the uterine wall and contrast medium leakage from the right posterior side of the uterine corpus to the cervix.

Based on these findings, we suspected hemorrhagic shock and DIC due to uterine rupture and decided to perform emergency laparotomy. On arrival, the levels of hemoglobin, platelets, and fibrinogen were 5.4 g/dL, 156,000/μL, and 126 mg/dL, respectively. Therefore, a total of 12 U of RBC, 6 U of FFP, and 3 g of fibrinogen were administered before surgery.

Surgery was initiated under general anesthesia, and the intraperitoneal space was found to be filled with a large amount of hemorrhage and blood clots. There was a 5‐cm laceration on the right posterior wall of the uterus, and since the laceration extended to the uterine artery and resulted in severe hemorrhage, we started hysterectomy for hemostasis. Six minutes after the start of the surgery, electrocardiogram showed ventricular tachycardia, which resulted in cardiopulmonary arrest.

**FIGURE 2:**
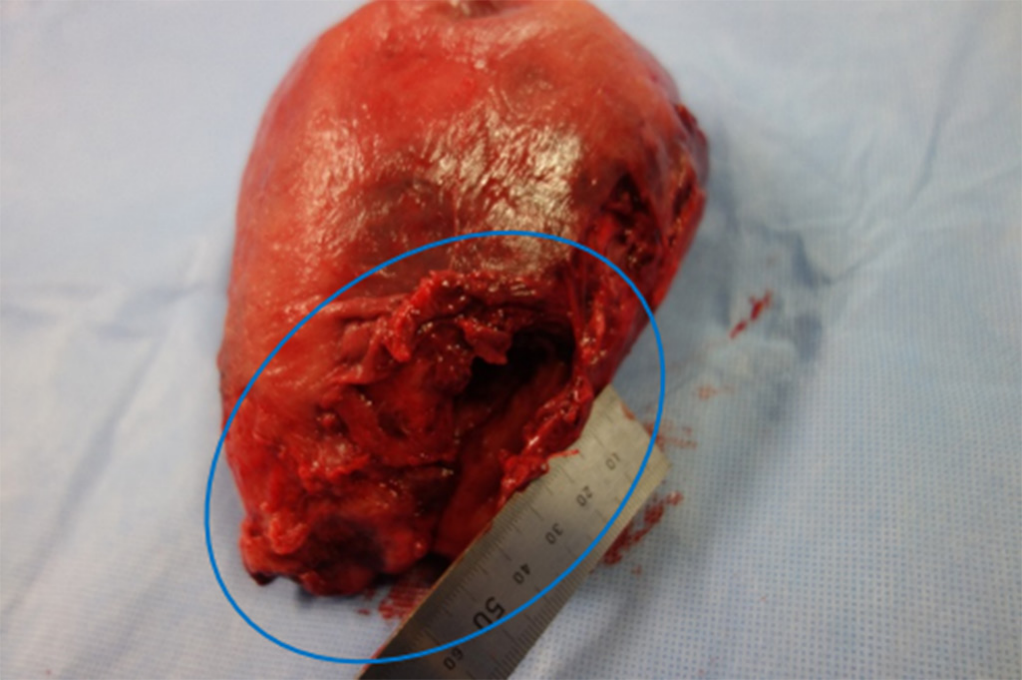
Removed uterine specimen. Uterus removed by supravaginal amputation. Rupture of the myometrium in the right posterior wall of the uterus (approximately 5 cm).

Cardiopulmonary resuscitation was performed with two defibrillations and five injections of adrenaline. Because arterial blood gas showed hyperkalemia (11.2 mg/dL) due to acidosis and rapid transfusion, it was treated with an injection of insulin and calcium gluconate hydrate. Because of the patient's cardiac arrest due to uterine rupture, we clamped the common iliac arteries bilaterally 10 min after the start of surgery and removed the uterus by supracervical hysterectomy 22 min after the start of surgery (Figure 2). Return of spontaneous circulation occurred 30 min after the start of surgery (cardiac arrest, 24 min).

Because of poor ventilation due to lung contusions from chest compressions and pulmonary edema due to rapid transfusion, we decided to introduce ECMO. Peripheral ECMO could not be performed following the clamping of the common iliac arteries; therefore, a cardiovascular surgeon opened the chest and introduced central ECMO (Venous‐Pulmonary Artery). After the introduction, the patient recovered ventilation.

After confirmation of intraperitoneal hemostasis, four drains were inserted bilaterally into the pleural spaces, mediastinum, and pelvic floor, and the surgery was completed. The total procedure time was 3 h 53 min, blood loss was 6790 mL, and blood transfusions were 3920 mL RBC, 1440 mL FFP, 400 mL platelet concentrate, and 7 g fibrinogen.

**FIGURE 3:**
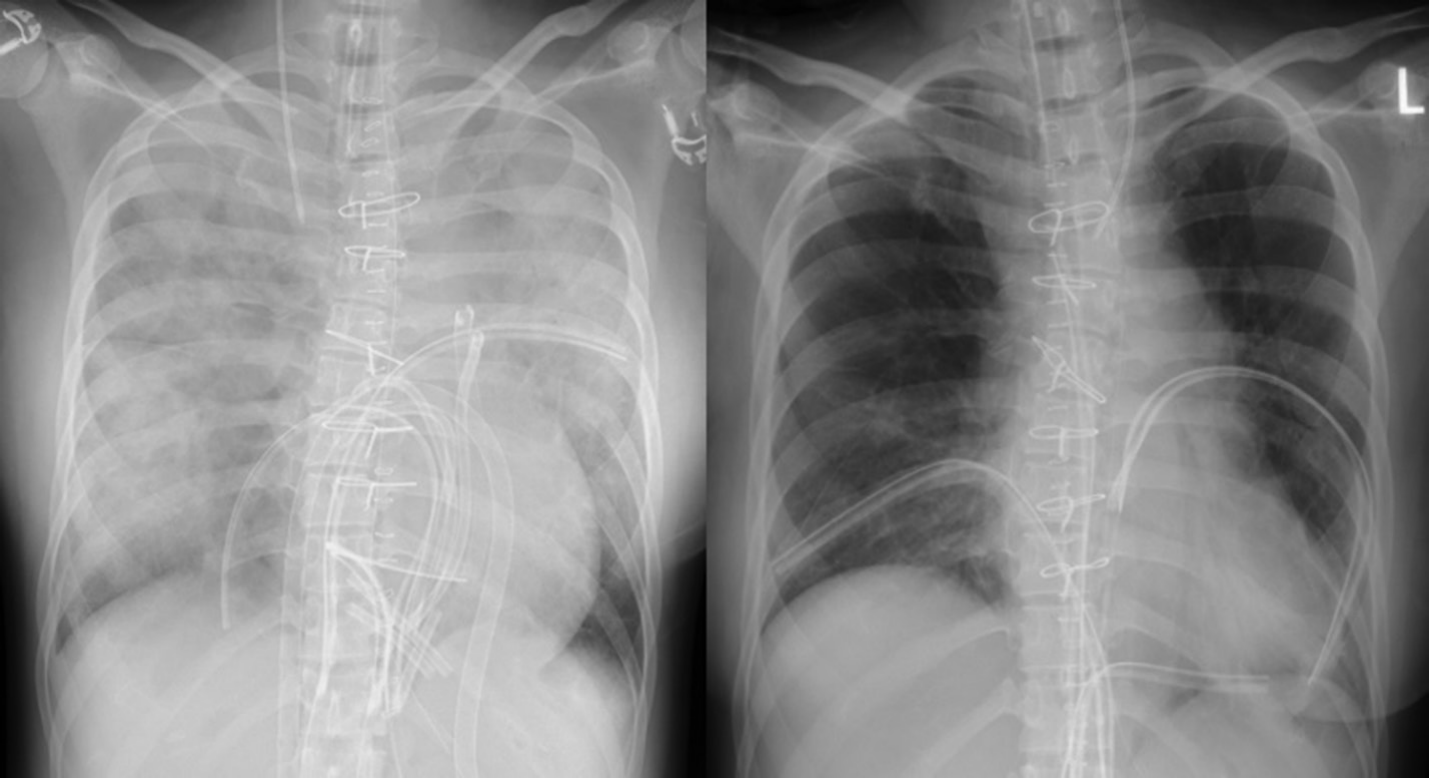
Chest radiograph at the introduction and weaning of ECMO. Lung field permeability improved. (Left: introduction, right: weaning).

After surgery, the patient was managed with ECMO in the ICU (intensive care unit) with continued anti‐DIC therapy. Chest radiography at the introduction of ECMO showed increased opacity of both lungs from the central to the peripheral regions, but no obvious fractures or hemopneumothorax. Lung rest using ECMO improved the PaO2 /FiO2 ratio in arterial blood and lung permeability (Figure 3). She was weaned from ECMO on the 7th postoperative day, and from the ventilator on the 9th postoperative day. She was transferred to a general ward and discharged home on the 21st postoperative day without any complications. No neurological symptoms or worsening of cardiac function was observed in the outpatient clinic after discharge.

## 3 DISCUSSION

In this report, we described a rare case of respiratory failure after resuscitation from cardiac arrest due to PPH complicated by DIC, which was successfully treated without any complications by ECMO with sufficient anti‐DIC therapy. This report showed the efficacy of introducing ECMO for respiratory failure even in cases of PPH complicated with DIC.

Maternal mortality rates are declining worldwide, decreasing by approximately 38%, averaging 216 per 100,000 from 2000 to 2017.^6^ However, the number varies depending on the country and region, and the number of maternal deaths in Japan is estimated to be 4–5 per 100,000 people,^7^ which is one of the lowest in the world. PPH is the most common cause of maternal death and cardiac arrest worldwide.^8^ In Japan, PPH is still a major cause of maternal death. PPH can cause hypovolemic shock and DIC, and rapid blood transfusion during treatment of PPH can cause pulmonary edema and cardiopulmonary failure.

In this case, the patient went into hemorrhagic shock due to uterine rupture and was administered a rapid blood transfusion because of DIC complications at the time of arrival at our hospital. As a result, she developed pulmonary edema and, at the same time, lung damage due to chest compressions for cardiac arrest, resulting in poor ventilation and requiring the introduction of ECMO. Lankford et al. reported 21 perinatal cases of ECMO induction. They reported that the indications included perinatal cardiomyopathy, septic cardiomyopathy, congenital heart disease, pulmonary thromboembolism, and ARDS.^9^

Currently, the number of ECMO introductions in perinatal cases is increasing; however, there are no previous reports of its use for lung contusions and pulmonary edema after cardiopulmonary resuscitation due to PPH comorbid with DIC. This is because its introduction may promote bleeding and DIC, as anticoagulants are used to prevent clots in the circuit, and platelet levels may decrease due to hemolysis caused by using the circuit^6^; this makes ECMO and DIC risk difficult to manage. The current case required ECMO introduction due to severe lung contusion and pulmonary edema, even though the patient had already developed severe DIC. Heparin was required to maintain the circuit of the ECMO. The dose was modified using the activated clotting time (ACT) as the index. The ACT was controlled at approximately 1.5 times the normal value, based on the guidelines.^5^ Adequate anti‐DIC therapy and blood transfusions of RBC, FFP, and fibrinogen prevented the complications of ECMO. Therefore, ECMO is may also an option for respiratory failure in patients with DIC if administered alongside sufficient anti‐DIC therapy.

In this case, the patient experienced cardiac arrest due to PPH with severe DIC, which was successfully treated with ECMO. However, it is also important to avoid conditions that require ECMO. For this purpose, it is necessary to establish a system that allows for appropriate delivery management and multidisciplinary collaborative treatment. In this case, the uterine rupture was caused by tachysystole secondary to the inadequate induction of labor and uterine fundal pressure during delivery. Therefore, labor induction should be conducted appropriately to prevent uterine rupture and PPH. Furthermore, a referral system should be established to facilitate the rapid transport of patients to higher‐level facilities and coordinate with multiple disciplines within the hospital. It has been reported that the delay between diagnosis and treatment plays a major role in maternal deaths in Japan.^10^ Our hospital has introduced a system called the “postpartum call system” to respond quickly to PPH cases that are transferred from other hospitals and to prevent the progression of severity in the maternal condition.^11^ This system allows information to be communicated to multiple departments, and the necessary medical resources to be used promptly. This system is effective for both the prevention and treatment of critically ill patients, which we believe is illustrated in this case, as the mother was saved, and complications such as hypoxic encephalopathy were prevented.

## 4 CONCLUSION

Although ECMO is relatively contraindicated in patients with PPH with severe DIC, it can be safely introduced by providing adequate anti‐DIC therapy through early multidisciplinary treatment. We believe that the construction of a system that enables early multidisciplinary treatment, which could provide advanced treatment including ECMO, will improve maternal prognosis.

## AUTHOR CONTRIBUTIONS

**Naoki Tsuchiya:** Writing – original draft. **Soichiro Obata:** Writing – original draft. **Michi Kasai:** Writing – review and editing. **Etsuko Miyagi:** Supervision; writing – review and editing. **Shigeru Aoki:** Supervision; writing – review and editing.

## FUNDING INFORMATION

This research did not receive any specific grant from funding agencies in the public, commercial, or not‐for‐profit sectors.

## CONFLICT OF INTEREST STATEMENT

The authors declare no conflicts of interest associated with this manuscript.

## CONSENT

Written informed consent was obtained from the patient to publish this report in accordance with the journal's patient consent policy.

## Data Availability

Data sharing is not applicable to this article as no new data were created or analyzed in this study.
